# Prodromal symptoms, health care seeking in response to symptoms and associated factors in eclamptic patients

**DOI:** 10.1186/s12884-017-1272-1

**Published:** 2017-03-14

**Authors:** Wondimu Gudu

**Affiliations:** Department of Obstetrics & Gynecology, Karamara General Hospital/Jigjiga University, P.O.Box: 238 Jigjiga, Somali Regional State Ethiopia

**Keywords:** Eclampsia, Prodromal, Precedent symptoms, Health care seeking, Factors affecting

## Abstract

**Background:**

Eclampsia is one of the leading causes of maternal death worldwide. Maternal catastrophe is made worse in developing countries by the high incidence coupled with delayed presentation of patients and health facility constraints in effective management of eclampsia and its complications.

**Methods:**

A prospective study of all 93 eclamptic women admitted to a general hospital in Somali regional state, Ethiopia was conducted between May 1, 2014 and April 30, 2015 using a structured questionnaire which included socio-demographic data, antenatal visit status, distance of nearest maternal health facility, timing of convulsions, questions related to symptoms preceding seizures; health care seeking for the symptoms and time interval from prodromal symptoms to the diagnosis of eclampsia. Descriptive statistics and multivariable logistic regression analyses were conducted. Statistical tests were done at a level of significance of P < 0.05.

**Results:**

There were 93 cases of eclampsia among 3500 deliveries with an incidence of 2.7%. The timing of Eclampsia was antepartum in 57 (61.3%); intrapartum in 26 (28.0%) and postpartum in 10 (10.7%). Most (63%) were not having any antenatal care (ANC) follow up. Precedent symptoms were reported in 73 (79.0%) of the mothers with severe head ache in 70 (75.0%); visual disturbance in 44 (47%) and epigastric pain in 17 (18.0%). The frequency of symptoms was not influenced by the timing of eclampsia and degree of hypertension and prodromal symptoms were reported in 80% of the patients with severe hypertension. The mean duration of prodromal symptoms before patients were diagnosed with eclampsia was 5.5 days. Only 19/73 (26.0%) of the patients with prodromal symptoms visited a health facility for their complaints prior to developing eclampsia. The diagnosis of hypertensive disorder of pregnancy was made in 8 (42.0%) of these patients. Independent predictors of failure to seek health care in response to preceding symptoms were: rural residence (*p*-value < 0.001) and distance of maternal health facility of > 5km (*p*-value < 0.01).

**Conclusions:**

Precedent symptoms were reported in most women. But many patients present late in response to these warning signs of eclampsia. Improving awareness of prodromal symptoms of eclampsia and timely health care seeking; providing ANC advises on danger signs of eclampsia in the socio-cultural context of the community; ensuring access to ANC services for rural mothers, and administration of anticonvulsants for all women with prodromal symptoms are recommended.

## Background

Eclampsia contributes to 18% of the 287 000 yearly maternal deaths globally [1]. However, the impact of the disease is felt more severely in developing countries where, unlike other more prevalent causes of maternal mortality (such as hemorrhage and sepsis), medical interventions may be ineffective due to late presentation of cases [2] (1^st^ delay in the 3-delays model of maternal mortality). Prompt health seeking behavior is essential because the reduction of the risk of death becomes more difficult when complications have developed [2].

To date, there are no reliable tests to predict preeclampsia/eclampsia [3] but eclampsia is mostly preceded by prodromal symptoms as has been reported in many retrospective studies. These precedent symptoms are nonspecific but may be surrogate markers of end organ dysfunction and disease severity and hence early warning signs of eclampsia [4]. The awareness of these symptoms by pregnant mothers will prompt timely health seeking behavior.

The incidence of eclampsia reported in the limited studies in Ethiopia varies from 0.3 to 0.7% [5, 6]. Eclampsia contributes to 11% of all maternal deaths and the case fatality rate is also high (3.6%) [7]. Only 3 - 18% of estimated eclamptic women attended emergency obstetric services [7]. But there are no studies done addressing factors associated with underutilization and delay in eclampsia care at any of the three levels of delay in maternal care.

This study is the first of its kind to characterize precedent symptoms of eclampsia prospectively in Ethiopia and the second in the literature [8]. The evaluation of precedent symptoms of eclampsia prospectively will provide more reliable incidence of symptoms than retrospective studies and sensitize health professionals on the clinical importance of the symptoms as early warning signs. Additionally, health care seeking of patients in response to precedent symptoms of eclampsia and the associated factors have never been studied. This will help to determine the timeliness of patients’ presentation and highlight areas of future intervention to address delay at individual level.

### Objectives

1. To evaluate prodromal symptoms in eclamptic women.
2. To assess health care seeking of patients in response to prodromal symptoms and timeliness of presentation after developing eclamptic seizures.
3. To assess factors associated with health care seeking of eclamptic patients in response to prodromal symptoms

## Methods

This was a prospective cross-sectional study conducted for one year (from May 2014 to April 2015) at Karamara General Hospital located in Jigjiga town of the Somali Regional State, in Eastern Ethiopia. Somali regional state is located 780 km from the capital of Ethiopia. Karamara hospital is the only regional hospital which also serves as the highest referral center for the region. The hospital provides a comprehensive emergency obstetric care with an estimated 3500 deliveries annually. Eclampsia is one of the most important complications of pregnancy that is a common picture in the OBGYN wards.

All 93 consecutive patients admitted to the obstetric & Gynecologic wards of Karamara hospital during the study period with convulsions and/or coma irrespective of gestational age as confirmed by senior Obstetricians were included. Eclampsia was defined as tonic-clonic seizures and/or unexplained coma during pregnancy (≥24weeks of GA) or postpartum with or without hypertension and/or proteinuria. All other medical causes were ruled out after appropriate clinical evaluation and laboratory tests/procedures including medical history for epilepsy; blood film for cerebral malaria; lumbar puncture for meningitis. But cranial imaging studies were not done because of facility constraints.

Data was collected using structured questionnaire by a single investigator. The questionnaire included information on socio-demographic data, the timing of convulsions, questions related to symptoms preceding seizures including headache, visual disturbance, epigastric pain; blood pressure at admission, health care seeking for the symptoms and time interval from onset of prodromal symptoms to the diagnosis of eclampsia.

Hypertension was defined as systolic blood pressure (BP) higher than 140 mm Hg, diastolic BP higher than 90 mm Hg, and severe hypertension was defined as a systolic BP higher than 160 mm Hg or diastolic BP higher than 110 mm Hg. Preeclampsia was considered in the presence of hypertension and proteinuria with or without edema. Chronic hypertension was defined as the presence of elevated BP (>140/90mmHg) predating or before 24weeks of pregnancy.

All eclamptic women were identified actively upon admission. Data was extracted both from direct interviewing of eclamptic women and/or their close attendants (in comatose patients) and from patients’ clinical records. Explanation was given about the study, verbal consent was obtained and anonymity in data collection assured. Ethical clearance for the conduct of the study was obtained from the health research ethics committee under Somali Regional state health Bureau.

Data was filtered, entered, and analyzed using EPI info version 7 statistical package. Frequencies, means, and tables were used for data summarization and presentation. Odds ratios (OR) and 95% confidence intervals (CI) were used to determine degrees of association. Multivariable Logistic regression analysis model was used to study the association between predictors (independent variables) and the binary dependent variable (health care visit). Predictor variables were selected based on literature data on health care utilization and common local practices.

## Results

There were 93 cases of eclampsia in 3500 deliveries during the study period with an incidence of 2.7%. Most were young (45% less than 20year) with a mean age of 23years..Seventy percent were nulliparous; 70.0% illiterate; 96% married and 67% residing out of the capital Jigjiga in. Most of the eclamptics (63.0%) were not having any Antenatal care (ANC) visits. The average gestational age of the pregnancies with antepartum/intra-partum eclampsia was 32.0week. Of these, 3.5% were previable (<28weeks); 62.3% preterm (<37weeks) and 34.1% term (≥37 weeks.). Gestational age was estimated both from clinical examination and ultrasound.

Most patients presented with convulsions in 84 (90.3%). Whereas, 9.7% of them presented with coma without prior report of seizures. The timing of Eclampsia was antepartum in 57 (61%); intrapartum in 26 (28.0%) and postpartum in 10 (11%). The duration of time lapsed from initial onset of convulsion/coma to patients presenting to the hospital ranged from 1 to 72 h, with a mean of 14.0 h.

Prodromal symptoms of eclampsia were reported in 73 (79.0%) of the patients. The most frequently reported symptoms were: severe headache in 70 (75%), visual disturbance in 44 (47%), and epigastric/right upper quadrant (RUQ) pain in 17 (18%). The occurrence (frequency) of the symptoms was higher in those with convulsion occurring before delivery compared to those with postpartum convulsions but the relative frequency of the symptoms was comparable in both groups [Table 1]

**Table 1:** Precedent symptoms of Eclampsia by timing of convulsions

Symptoms	Ante/intrapartum (*n* = 83)	Post partum (*n* = 10)	Total (*n* = 93)
Severe Head ache	64 (77.0%)	6 (60%)	70 (75.0%)
Visual disturbance	41 (49.0%)	3 (30%)	44 (47.0%)
Epigastric/RUQ pain	15 (18.0%)	2 (20%)	17 (18.0%)
Nausea/vomiting	8 (10%)	1 (10%)	9 (10.0%)

The interval from initial onset of precedent symptoms to the diagnosis of eclampsia at a health facility ranged from 1 to 15 days with a mean of 5.5 days. Almost two-thirds (65%) of the cases had precedent symptoms lasting for at least 7 days prior to being diagnosed with eclampsia [Table 2].

**Table 2:** Duration of precedent symptoms before the diagnosis of Eclampsia

Duration of precedent symptoms	Frequency	Percent	Cum. percent
1 to 3 days	21	28.8%	28.8%
4 to 6 days	22	30.1%	57.5%
7 to 9 days	27	37.0%	95.9%
10 to 15days	3	4.1%	100.00%
Total	73	100.00%	100.00%

The majority (96%) patients with prodromal symptoms had hypertension at admission with severe hypertension (DBP ≥ 110mmhg) in 52/73 (71%) and moderate hypertension (DBP 100–110) in 11/73 (15%) of the patients respectively. Conversely, 49/61 (80%) of the patients with severe hypertension and 17/23 (74%) of the eclamptics with moderate hypertension reported one or more Prodromal symptoms. (Table 3)

**Table 3:** Preceding Symptoms of Eclampsia Vs Degree of Hypertension

Symptom/Sign	Severe Hypertension (*n* = 61)	Moderate Hypertension (*n* = 23)
Head ache	46 (75%)	17 (74%)
Visual disturbance	32 (52%)	10 (43%)
Epigastric/RUQ pain	9 (15%)	3 (13%)
Vomiting	5 (8%)	1 (4%)
Asymptomatic	11 (18%)	6 (26%)

Only 19 (26.0%) of the patients with one or more prodromal symptoms visited a health facility for their complaints prior to developing eclampsia. A clinical diagnosis of hypertensive disorder of pregnancy was made in 8 (42.0%) of these patients. All had the diagnosis made 7 days prior to developing eclampsia with a mean of 9.0 days and range of 7 to 25 days. Six had preeclampsia; one had superimposed preeclampsia and one patient had chronic hypertension.

Considering the fact that prodromal symptoms of eclampsia would prompt health care visit; Socio-demographic and distance factors associated with health care seeking of patients in response to prodromal symptoms were assessed and the findings are summarized in table 4. Failure to seek health care for precedent symptoms was more likely in rural residents (OR = 12.0, 95% CI [3.6, 42]); in those who are illiterate (OR = 3.5, 95% CI [1.1, 11]) and in mothers living >5km away from a maternal health facility (OR = 4.0, 95% CI [3.9, 11]). But on multivariable logistic regression analysis, only place of residence (*p*-value 0.001) and distance of the nearest health facility from patients’ home (*p*-value = 0.01) were found to be independent predictors of health care seeking.

**Table 4:** Association between predictors and health care visit for prodromal symptoms based on multivariable logistic regression analysis

Predictors	No Health care visit	Health care visit			
	(*n* = 54)	(*n* = 19)	Crude OR	* Adjusted OR	*p*-value
	Number (%)	Number (%)	(95% ** CI)	(95% CI	
Adress					
Out of Jijiga	44 (90%)	5 (10.0%)			
^Rc^ Jijiga	10 (42%)	14 (59.0%)	12.3 [3.6, 42]	27.6 [4.5, 170]	0.001
Age					
<20 (*n* = 33)	26 (79%)	7 (21%)			
^Rc^ > 20 (*n* = 40)	28 (70%)	12 (30%	1.6 [0.5, 4.7	1.1 [0.25, 4.9]	0.9
Ethnicity					
Somali (*n* = 69)	51 (74%)	18 (26.0%)	0.9 [0.09, 10.0]	1.9 [0.01, 38.6]	0.67
^Rc^ Oromo (*n* = 4)	3 (75%)	1 (25%)			
Marital status					
others (*n* = 7)	5 (71%)	2 (29.0%)	0.9 [0.2, 5]	1.9 [0.1, 38.3]	0.68
^Rc^ Married (*n* = 66)	49 (74%)	17 (26%.)			
Literacy					
Illiterate (53)	43 (81%)	10 (19.0%)	3.5 [1.1, 11]	1.2 [0.3, 5]	0.75
^Rc^ Literate (20)	11 (55%)	9 (45%)			
Occupation					
Unemployed (63)	48 (76%)	15 (24%)	2. [0.5, 9]	3.2 [0.4, 23.0]	0.26
^Rc^ Employed(*n* = 10)	6 (60%)	4 (40%)			
Parity					
Nullipara (*n* = 51)	38 (75%)	13 (25%)	1 [0.4, 3.4]	0.7 [0.15,3.7]	0.7
^Rc^Multlipara (*n* = 22)	16 (73%)	6 (27%)			
Distance of health care					
>5km (*n* = 59)	47 (78%)	12 (22.0%)	3.9 [1.2, 14]	14.8, [1.8, 18]	0.01
^Rc^ < 5km (*n* = 14)	7 (57%)	7 (43.0%)			
ANC follow up					
No (*n* = 27)	19 (70%)	8 (30%)	0.7 [0.3, 2.1]	0.5 [0.1, 2]	0.3
^Rc^Yes (*n* = 46)	35 (76%)	11 (24%)			

* *AOR (Odds Ratio)* Adjusted for all other variables in the table, ** *CI* Confidence Interval, * *Rc* Reference category

The mean distance of the nearest health facility providing maternal services (including delivery) from patient’s home was 9kms (range 1–30 km). Most (52%) of the mothers were living more than 5km from these facilities.

Magnesium Sulfate was administered to all eclamptic patients except a single woman. Whereas, Hydralazine for acute control of hypertension was used in 70/93 (76%) of the cases.

The cause-specific maternal death rate for eclampsia was 285 per 10,000 deliveries. Eclampsia accounted for (proportionate mortality rate) 29% of all maternal deaths and the case fatality rate was 11%. The gross perinatal mortality rate was 26/75 (346 per 1000 deliveries).

## Discussion

The incidence of eclampsia (2.7%) was high compared to reports from hospital based studies in the same country and most other African countries [5], [6], and [7]. Eclampsia remains the leading cause of death in the study area contributing to 29% of all maternal deaths. Hence, the early detection and timely management of eclampsia are crucial to mitigate the impact of eclampsia on maternal health in the study area. To this end, symptoms and signs of preeclampsia are important as early warning signs and are presumed to be better than laboratory investigations to predict adverse maternal outcomes including eclampsia [9].

Prodromal symptoms of eclampsia are a result of generalized vasospasm, fibrin and platelet deposition, and occlusion of blood flow to vital organs [8]. Prodromal symptoms were mostly studied retrospectively which were prone to bias and the reported incidences vary from 41 to 91% [8]. The frequency of prodromal symptoms in this study (79%) is comparable to that of the 83% and 90% rates reported in the two studies done in Tanzania which characterized prodromal symptoms prospectively [8, 10].

Headache and visual disturbance reflect cerebral edema and vasospasm of cerebral and retinal vessels. These neurological symptoms were the most commonly reported symptoms of imminent eclampsia in all retrospective studies done in similar settings which range from 75 to 100% for headache and 10 -21% for visual disturbance [11–13]. The corresponding rates were 80% and 45% respectively in the prospective study done in Tanzania [8] and 88% and 39% in the other case-referent study from the same country [10]. Our findings (75% for headache and 47% for visual disturbance) were comparable to most studies except a higher incidence of visual disturbance compared to the findings from retrospective studies [11–13].

Epigastric/RUQ pain implies sub-capsular hemorrhage, necrosis and edema of the liver [9, 10]. The frequency of epigastric pain (18%) in this study was higher than most reports but comparable to the 20% incidence in the Tanzanian study [8] and lower than the 36% in the other case-referent study [10].

Although there are numerous literature data on the frequency and characteristics of prodromal symptoms of eclampsia, studies on the predictive value of these symptoms are limited. A review of women with preeclampsia or HEELP syndrome gave an odds ratio of 3.6 for headache to predict eclampsia [8]. The other recently published PIERS model gave epigastric pain an odds ratio of 2.92 for predicting adverse outcomes in preeclampsia [10].

Although the design of this study couldn’t allow the development of any predictive model, the following findings of the research suggest prodromal symptoms can be used as early warnings signs of eclampsia in the study area: Most (79%) of the patients with eclampsia had prodromal symptoms; the presence of prodromal symptoms was associated with hypertension in 96% of the cases (71% with severe hypertension); prodromal symptoms were reported in 80% of patients with severe hypertension; and importantly the relative frequency of symptoms was not affected by timing of eclampsia and degree of hypertension (e.g. headache: 80% in those with severe hypertension and 74% in those with moderate hypertension).

But the importance of prodromal symptoms should not be overemphasized as the symptoms are non-specific and eclampsia can develop without prodromal symptoms in some women (21% in our study). Hence, clinicians should always consider other medical diagnoses in the evaluation of women with symptoms such as severe headache, blurring of vision and nausea/vomiting during or shortly after pregnancy.

The findings of this study on prodromal symptoms will be even more pronounced in the face of many researches that show prodromal symptoms have not been given due attention as important danger signs by both health professionals and pregnant mothers in Ethiopia. In recent studies, only 1/3rd of pregnant mothers were aware of danger signs of pregnancy: 39% in Debre Birhan [14]; 32% in Ambo [15] and 27% in Goba [16]. Awareness of premonitory symptoms of eclampsia ranged from 8% for blurring of vision to 52% for headache [14], [15], and [16]. The study on the performance of HEW in Tigray (2012) showed that their knowledge on prodromal symptoms of eclampsia was very poor with 30% for severe headache, 12% for visual disturbance and 0% for epigastric pain [17]. Hence, the importance of prodromal symptoms as early warning signs of eclampsia should be re-emphasized to all health providers (especially front line) as well as pregnant mothers and awareness to the symptoms should be raised. There should also be a consideration to use prodromal symptoms for screening/triage of pregnant mothers for referral by health extension workers (HEW) in Ethiopia. The recent effective model which introduced a simple patient-held pictorial card reminding women of the symptoms of imminent eclampsia and the actions to take could be adapted in the study area to be used by community health workers and lower level health facilities [7, 18].

The timeliness of patients’ presentation in response to prodromal symptoms of eclampsia and/or after they develop convulsions/coma is important in the effective management of eclampsia and its complications. Overall the presentation of patients in this study was late (delayed) as the mean duration of onset of coma/convulsions before patients presented to the referral hospital was 13 h. The health care seeking of patients in response to precedent symptoms of eclampsia was also low as 54 (74%) of the patients didn’t visit any health facility despite experiencing imminent symptoms of eclampsia for an average of 5.5 days. Our findings strengthen the previously reported very low utilization of emergency obstetric services by eclamptic women in Ethiopia [7]. This is of concern as the presence of prodromal symptoms is expected to prompt patients to seek timely health care. In the absence of reliable predictive tests and health facility constraints in the effective management of eclampsia, the reduction in the high incidence of eclampsia and subsequent maternal mortality in the study area much depends on the timeliness of patients presentation coupled with timely provision of eclampsia care.

There may be socio-cultural and health facility factors that may affect health care seeking of patients (other than individual factors) including acceptability, perceived quality of care, distance of maternal care facility, service fees, alternative(cultural) remedies [19]. Hence, identifying all the factors associated with delayed presentation of eclamptic patients is of paramount importance. In this study, independent predictors of not seeking care were: place of residence (*p*-value < 0.001) and distance of maternity health care of >5km (*p*-value = 0.01). This finding is in agreement with other research evidence of a huge Urban–rural disparity in Antenatal care utilization in Ethiopia [20]. Hence maternal health service provision should be targeted to address this disparity and ensure access to ANC services for the underprivileged rural mothers. Interestingly, ANC follow up didn’t affect health care seeking significantly. This has implication on the content and quality of ANC service provision in the area including lack of knowledge on the provider side; lack of proper advice on danger signs and where to seek help and possible patient-provider communication barriers.

Only Less than half (42%) of the patients who presented to a health facility with prodromal symptoms (before developing eclampsia) were diagnosed with HDP. This suggests there might have been health facility/provider failures in proper evaluation & early diagnosis of HDP. There is scientific evidence that prodromal symptoms of eclampsia are mostly associated with hypertension. Hence, capacity building of the facilities (especially lower level) through periodic refreshment on-job training of health professionals and provision of basic equipments used for diagnosing hypertension should be considered.

Based on observations from clinical practice, there is a general tendency among health providers of withholding the administration of prophylactic anticonvulsant in women presenting solely with prodromal symptoms without associated severe hypertension. Considering the consistently high incidence of precedent symptoms irrespective of the timing of eclampsia and severity of hypertension, the universal administration of prophylactic anticonvulsants in all women with prodromal symptoms should be emphasized in eclampsia management guidelines.

There are some limitations of the study. The absence of controls (normotensive mothers) might weaken recommendation of utilizing prodromal symptoms as reliable warning signs of eclampsia for the general obstetric population. The validation of symptoms is also influenced by recall bias as patients were asked for symptoms only after they develop eclampsia. To reduce recall bias data was collected by direct interviewing of patients immediately upon admission and it is believed that the short duration of most symptoms would allow patients to memorize symptoms easily. Barriers to health care seeking in women with prodromal symptoms/eclampsia at all levels (including health facility, accessibility) were not addressed comprehensively and hence future studies addressing all the factors are recommended.

## Conclusions

Precedent symptoms are reported by most women with eclampsia and can be used as warning signs of imminent eclampsia. But most patients present late in response to these symptoms, predominantly those residing in rural areas. Capacity building of health professionals to improve their knowledge on precedent symptoms of eclampsia; and timely diagnosis of patients presenting with precedent symptoms; educating mothers on danger signs of eclampsia in the socio-cultural context of the community with emphasizing timely health care seeking and ensuring access to ANC services for the underprivileged rural mothers are recommended. The universal use of prophylactic anticonvulsants in women with prodromal symptoms irrespective of the severity of hypertension should be emphasized; Further large-scale studies to identify all the factors associated with the delay in seeking maternal health care in eclamptic women should be conducted as well as well-designed studies to determine the predictive value of prodromal symptoms of eclampsia.

## Abbreviations

ANC: Antenatal care
DBP: Diastolic blood pressure
HDP: Hypertensive disorders of pregnancy
HEW: Health extension workers
RUQ: Right upper quadrant
SBP: Systolic blood pressure

## References

[1] World health organization. World Health Statistics 2012: WHO 2012. www.who.int/gho/publications/world_health_statistics/2012/en/.

[2] Kayode O. Osungbade and Olusimbo K. Ige. Public Health Perspectives of Preeclampsia in Developing Countries: Implication for Health System strengthening. Hindawi Publishing Corporation Journal of Pregnancy. Volume 2011, Article ID 481095, 6 pages doi:10.1155/2011/481095 10.1155/2011/481095 PMC3087154 21547090

[3] Thangaratinam S, Landegveld J, Mol BW, Khan KS (2011). Prediction and primary prevention of preeclampsia. Best Prect Res Clin Obstet Gynaecol.

[4] Thangaratinam S (2011). How accurate are maternal symptoms in predicting impending complications in women with preeclampsia? A systematic review and meta-analysis. Acta Obstet Gynaecol Scand.

[5] Mekbib TY, Ketsela K (1991). Preeclampsia/eclampsia at Yekatit 12 hospital Addis Ababa. EAMJ.

[6] Abate M, Lakew Z. Eclampsia a 5 years retrospective review of 216 cases managed in two teaching hospitals in Addis Ababa. Ethiop Med J. 2006;44(1):27–31. 17447360

[7] Gaym A, Bailey P, Pearson L, Admasu K, Gebrehiwot Y (2011). Disease burden due to pre-eclampsia/eclampsia and the Ethiopian health system’s response. Int J Gynaecol Obstet.

[8] Cooray SD, Edmonds SM, Tong S, Samarasekera SP, Whitehead C (2011). Characterization of symptoms immediately preceding eclampsia. Obstet Gynecol.

[9] Cavkaytar S (2017). Are clinical symptoms more predictive than laboratory parameters for adverse maternal outcome in HEELP syndrome?. Acta Obstet Gynaecol Scand.

[10] France J, Muganyizi PS (2012). Characteristics of symptoms of imminent eclampsia: A case referent study from a tertiary hospital in Tanzania. Open J Obstet Gynecol.

[11] Onuh SO, Aisien AO (2004). Maternal and fetal outcome in eclamptic patients in Benin City, Nigeria. J Obstet Gynaecol.

[12] Onwuhafua PI, Onwuhafua A, Adze J, Mairami Z (2001). Eclampsia in Kaduna State of Nigeria--a proposal for a better outcome. Niger J Med.

[13] Okogbenin SA (2010). Eclampsia in Irrua Specialist Teaching Hospital: a five-year review. Niger J Clin Pract.

[14] Solomon AA, et al. Knowledge about danger signs of pregnancy and associated factors among pregnant women in Debra Birhan Town, Central Ethiopia. Sci J Public Health. 2015;3(2):269–73. Published online March 11, 2015 (http://www.sciencepublishinggroup.com/j/sjph) doi:10.11648/j.sjph.20150302.27. Accessed 12 Feb 2016.

[15] Yabo AN, et al. Assessment of Quality of Antenatal Care (ANC) service provision among pregnant women in Ambo Town Public Health Institution, Ambo, Ethiopia, 2013. Am J Nurs Sci. 2015;4(3):57–62. Published online April 18, 2015 (http://www.sciencepublishinggroup.com/j/ajns) doi:10.11648/j.ajns.20150403.13. Accessed 12 Feb 2016.

[16] Bogale D, Marko D (2015). Knowledge of obstetric danger signs among child bearing age women in Goba district, Ethiopia: a cross sectional study. BMC Pregnancy Childbirth.

[17] Medhanyie A, et al. Knowledge and performance of the Ethiopian health extension workers on antenatal and delivery care: a cross-sectional study. Hum Resour Health. 2012;10:44. http://www.human-resources-health.com/content/10/1/44. Accessed 12 Feb 2016. 10.1186/1478-4491-10-44 PMC3536599 23171076

[18] MacGillivray I, McCaw-Binns AM, Ashley DE, Fedrick A, Golding J (2004). Strategies to prevent eclampsia in a developing country: II. Use of a maternal pictorial card. Int J Gynecol Obstet.

[19] Inem VA (2010). Non-uptake of facility-based maternity services in an inner-city community in Lagos, Nigeria: an observational study. J Biosoc Sci.

[20] Yesuf EA, Calderon-Margalit R (2013). Disparities in the use of antenatal care service in Ethiopia over a period of fifteen years. BMC Pregnancy Childbirth.

